# Supplementary material for: SARS-CoV-2 Evolution among Oncological Population: In-Depth Virological Analysis of a Clinical Cohort
Source: Microorganisms. 2021 Oct 14;9(10):2145. doi: 10.3390/microorganisms9102145 (PMC8540785; doi:10.3390/microorganisms9102145)
Supplement: Supplementary file 1 [file microorganisms-09-02145-s001.zip › microorganisms-1399544-SI.pdf]

Table S1: Patients' baseline characteristics

	Total<br>n=54
**Demographics**	
Sex (male), n (%)	31 (57.4)
Age, mean (sd)	67.2 (14.1)
**Oncological diagnosis, n (%)**	
**Solid**	35 (64.8)
Lung	6 (11.1)
Prostate	9 (16.7)
Gynecologic	7 (13.0)
Other	13 (24.1)
**Onco-hematological, n (%)**	20 (37.0)
LLC	8 (14.8)
Lymphoma	4 (7.4)
Monoclonal gammapathy	3 (5.6)
MDS	3 (5.6)
Other	2 (3.6)
**Disease stade, n (%)**	
Localized	33 (61.1)
Disseminated	21 (38.9)
**Active treatment, n (%)**	29 (53.7)
Allogeneic stem cell transplantation	3 (5.6)
Chemotherapy	15 (27.8)
Steroids	8 (14.8)
Surgery	6 (11.1)
Radiotherapy	8 (14.8)
Other	14 (25.9)
**Comorbidities, n (%)**	
Pulmonary disease	7 (13.0)
Hypertension	18 (33.3)
Diabetes	6 (11.1)
Obesity	18 (33.3)

Smoking	7 (13.0)
**Performance score, ECOG, n (%)***	
**0**	21 (70.0)
**1**	6 (20.0)
**2**	2 (6.7)
**3**	1 (3.3)
**Laboratory tests, median, (IQR)**	
Lymphocytes	1 (1.2)
Thrombocytes	185 (126)
Neutrophils	4.3 (5.1)
Monocytes	0.8 (0.1)
CRP	95.0 (90.7)
LDH	297.5 (221)

One patient had solid and onco-hematological cancer. Other onco-hematological disorders include Vazquez and Castleman diseases. Other treatments include immunotherapy, tyrosine kinase inhibitors, monoclonal antibodies and hormonotherapy. *information available for 30 patients

Table S2: Patients' COVID disease characteristics

	**Total**<br>n=54
**Delay from SO to diagnosis, median (IQR)**	3 (6)
**Symptoms, n, (%)**	**46**
Fever	30 (65.2)
Cough	26 (56.5)
Shortness of breath	11 (23.9)
Fatigue	7 (15.2)
Rhinorrea	6 (13.0)
Myalgia	4 (8.7)
Digestive symptoms	3 (6.5)
Shivering	3 (6.5)
Sore throat	2 (4.3)
Anosmia, dysgueusia	2 (4.3)
Headache	2 (4.3)
**Hospitalization, n (%)**	36 (78.3)
**Delay from diagnosis to hospitalization, median (IQR)**	1 (3.3)
**Length of stay, median (IQR)**	15.5 (15.5)
**Affected organ, n (%)**	**32**
Lower respiratory tract	30 (93.8)
Nervous system	1 (3.1)
Digestive system	5 (15.6)
Kidneys	4 (12.5)
Cardiovascular	4 (12.5)
**Co-infections, n (%)**	
Bacterial	3 (5.6)
**Treatment, n (%)**	
Oxygen	28 (51.9)
Steroids	8 (14.8)
Remdesivir	4 (7.4)
**Intubation, n (%)**	7 (12.9)
**Intubation length, median days (IQR)**	12 (7)
**Death, n (%)**	12 (22.2)

Symptoms were missing for 8 patients, proportion was calculated with a total of 46; Affected organ was available for 32 patients; SO: symptom onset; IQR: interquartile range; among bacterial co-infection, 2 *S pneumoniae* and 1 *H Influenza*

Table S3: Minority variants summary Table

ID patients	Minority variant nucleotide position	%	Region	Minority variant amino acid change
2a	C17010T	9,63%	ORF1ab	No
2b	C17010T	32,32%	ORF1ab	No
4a	C15324T	0,50%	ORF1ab	No
4b	C15324T	22,11%	ORF1ab	No
4a	T29685C	37,74%	Untranslated	No
4b	T29685C	6,06%	Untranslated	No
5a	C26333T	0,41%	E gene	T30I
5c	C26333T	40,10%	E gene	T30I
6a	T12669C	15,00%	ORF1ab	V4136A
6b	T12669C	38,97%	ORF1ab	V4136A
6a	G19802T	41,03%	ORF1ab	R6513L
6b	G19802T	0,09%	ORF1ab	R6513L
